# Relationship Between Immunohistochemical CD3, CD4, CD5, CD8, and PD1 Staining and Histopathological Diagnosis of Cervical Lesions in Patients With Abnormal Colposcopic Findings

**DOI:** 10.7759/cureus.31399

**Published:** 2022-11-12

**Authors:** Ayhan Atigan, Tolga Güler, Yeliz Arman karakaya, Derya Kilic

**Affiliations:** 1 Gynecology and Obstetrics, Karabuk University, Medicine Faculty, Karabük, TUR; 2 Obstetrics and Gynecology, Pamukkale Unıversity, Ankara, TUR; 3 Pathology, Pamukkale Unıversity, Denizli, TUR; 4 Obstetrics and Gynecology, Pamukkale University, Denizli, TUR

**Keywords:** immunohistochemical staining, abnormal colposcopic finding, tumor infiltrating lymphocyte, cervical intraepithelial neoplasia, squamous intraepithelial lesion

## Abstract

Introduction: This study aimed to analyse the relationship between clinicopathological factors in cervical intraepithelial lesions and abnormal colposcopic findings.

Material and methods: Thirty high-grade squamous intraepithelial lesion (HSIL) and thirty low-grade squamous intraepithelial lesion (LSIL) patients who underwent biopsy due to abnormal colposcopic findings were included in the study. The immunoreactivity of CD3, CD4, CD5, CD8, and PD-1 was analysed immunohistochemically in tumor-infiltrating lymphocytes (TILs) and stromal lymphocytes.

Results: In TILs, CD3, CD4, CD5, CD8, and PD-1 were highly stained in 20/30 (66.6%), 16/30 (53.3%), 15/30 (50.0%), 24/30 (80.0%), and 13/30 (43.3%) of the cases for the HSIL group, while 7/30 (23.3%), 4/30 (13.3%), 5/30 (16.6%), 9/30 (30.0%), and 5/30 (16.6%) were in the LSIL group, respectively. CD3, CD4, CD5, CD8, and PD-1 immunostainings for TILs were higher in the HSIL group (p=0.001, p=0.001, p=0.006, p˂0.001, p=0.024, respectively). Only PD-1 was significantly higher in lymphocytes in the stroma (p=0.001).

Conclusions: CD3, CD4, and CD8 also show a positive correlation with the Ki-67 proliferation index. CD3, CD4, CD5, and CD8 may contribute to PD-1-mediated tumour control. Immunohistochemical staining plays a key role in evaluating the tumour microenvironment.

## Introduction

Cervical cancer remains one of the most common causes of death for women, although its incidence has been reduced by screening tests [1]. According to Globocan 2012 data published by the International Cancer Agency (IARC), cervical cancer ranks fourth among all cancers in women and is the most common among female genital organ cancers [2]. It differs from other cancers because it has a screening program and is a preventable cancer. The human papilloma virus (HPV) is effective as the cause of abnormal cytological findings in cervical cancer and cervical preinvasive lesions. Despite the high lifetime probability of HPV infection, the infection often regresses spontaneously. However, when HPV persists, it causes cervical intraepithelial neoplasia (CIN) [low-grade squamous intraepithelial lesion (LSIL) (CIN 1) and high-grade squamous intraepithelial lesion (HSIL) (CIN 2 and CIN 3)] before cervical cancer [3]. The use of HPV DNA tests and simultaneous pap smear tests is called co-test and is the current approach for cervical cancer screening programs. Co-test screening is recommended every five years to women over 30 by the ASCCP (American Society of Colposcopy and Cervical Pathology), ACS (American Cancer Society), ACOG (American Academy of Gynecology and Obstetrics), and USPSTF (US Preventive Services Task Force) [4]. Turkey has become the leading country in Europe for cervical cancer screening with its HPV-based screening program spread throughout the country [5].

The stage of colposcopic examination is initiated based on the results of the co-test performed during cervical cancer screening. The colposcopy-guided biopsy specimen is accepted as the gold standard method in the diagnosis of cervical intraepithelial lesions [6]. Higher colposcopy referral rates have coincided with the HPV-based screening program. However, detection rates of high-grade lesions (CIN2 and CIN3) have increased [5].

The immune system is thought to play an important role in carcinogenesis, particularly in the prevention of tumor development. In various types of cancer, increased tumor-infiltrating lymphocyte (TIL) concentration is associated with a favorable prognosis [7]. Recent advancements in immunotherapy, particularly immune checkpoint inhibitors (ICIs), have heightened interest in the new treatment strategy as well as the immune status of the cancer microenvironment [8]. Tumor cells modify the tumor microenvironment to both suppress T cells and induce tumorigenic inflammation. PD-1, also called CD279, which has two ligands, inhibits T-lymphocyte functions with the combination of these ligands (PD-L1 [CD274] and PD-L2 [CD273]) [7,9]. Survival in recurrent cervical cancer patients with the programmed cell death 1 (PD-1)-blocking antibody Cemiplimab, which is used in lung and skin cancers, was significantly longer than with single-agent chemotherapy [10]. TILs such as CD3, CD4, CD5, and CD8, which attack cancer cells directly or contribute to antigen presentation, prevent tumor development in carcinogenesis [7,11,12].

The goal of this study is to examine the relationship between clinicopathological factors in cervical intraepithelial lesions, such as the proliferation of PD-1+, CD8+, CD5+, CD4+, and CD3+ T lymphocytes infiltrating the tumor and tumor stroma, and the degree of the lesion in cases with abnormal colposcopic findings.

## Materials and methods

Patients

Patients admitted to the cervical lesion unit of a tertiary center were screened through the hospital information system using a retrospective study design. Sixty cases diagnosed with cervical intraepithelial lesions by colposcopy-guided excision procedures between January 01, 2015, and November 01, 2019 were included in the study. Thirty of these sixty cases are HSILs, and thirty are LSILs.

Colposcopic examination and excision procedures were performed as described in our recent article by recommended guidelines [3]. Colposcopy can reveal abnormal findings such as acetic white epithelium, leukoplakia, atrophic changes, punctated blood vessels, mosaics, and white rings around gland openings. Patients with abnormal findings in the colposcopic examination were included, while patients whose examination could not be completed or who had previously known cervical dysplasia were excluded from the study.

The Non-Interventional Clinical Research Ethics Committee of Pamukkale University Medicine Faculty gave ethical approval for the study (decision number: 19/11/2019-20).

Immunohistochemistry

**Figure 1:**
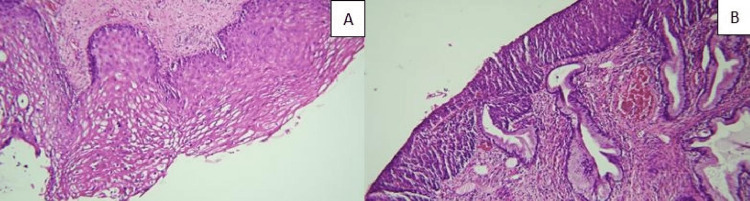
(A) CIN I, H&E, 100× and (B) CIN III, H&E, 100×. CIN: cervical intraepithelial neoplasia H&E: hematoxylin and eosin.

Ki-67 and hematoxylin-eosin (H&E) (Figure 1A-1B) stained sections were reexamined and the areas of the lesions were marked. Cervical intraepithelial lesion paraffin blocks were chosen for immunohistochemical examination, and sections of an appropriate thickness (approximately 3-5 µm) were prepared. These sections, which were placed on a positively charged glass slide, were studied with the Ventana Benchmark XT™ or Ventana Benchmark ULTRA™ fully automatic stainers using the ultraView Universal DAB detection kit for all staining. Immunohistochemical staining was accomplished using antibodies of CD3 (Ventana, prediluted-auto-ready-to-use antibody; CONFIRM anti-CD3 (2GV6) Rabbit Monoclonal Primary Antibody, Tucson, Arizona 85755, USA); CD4 (Ventana, prediluted auto-ready-to-use antibody; CONFIRM anti-CD4 (SP35) Rabbit Monoclonal Primary Antibody, Tucson, Arizona 85755, USA); CD5 (Ventana, prediluted auto-ready-to-use antibody; CONFIRM anti-CD5 (SP19) Rabbit Monoclonal Primary Antibody, Tucson, Arizona 85755, USA); CD8 (Ventana, prediluted auto-ready antibody; CONFIRM anti-CD8 (SP57) Rabbit Monoclonal Primary Antibody, Tucson, Arizona 85755, USA); PD1 (CD279) (Cell Marque, prediluted-auto-ready-to-use antibody; Mouse monoclonal, clone: NAT105, Rocklin, CA 95677, USA). The primary antibody step was skipped for the negative control in immunohistochemical staining. Examined areas on the slide were evaluated by at least two researchers, one of whom was a pathologist, with a Nikon Eclipse e200 microscope. Tonsil tissues were used as instructed for all stainings for positive control. Cytoplasmic and/or membranous staining in tumor-infiltrating lymphocytes and stromal lymphocytes were considered for the immunoreactivity of CD3, CD4, CD5, CD8, and PD-1. They were stated the percentage ratio of CD3, CD4, CD5, CD8, and PD-1 T lymphocytes in lymphocytes infiltrating the tumor (cervical intraepithelial lesion area in epithelium) and stroma. In the epithelium (intratumoral) percentages greater than 20%, 10%, 10%, 10%, and 1% for CD3, CD4, CD5, CD8, and PD-1, respectively, were considered high, while smaller percentages were considered low. Percentages greater than 40%, 20%, 20%, 20%, and 1% for CD3, CD4, CD5, CD8, and PD-1 in the stroma, respectively, were considered high, while smaller percentages were considered low.

Statistical analysis

The descriptive values of quantitative continuous variables (such as age) were analyzed using standard descriptive statistical methods (such as the arithmetic mean, standard deviation, and median). Categorical variables (presence frequencies) are given with their frequencies and percentages in the total. Quantitative measurements were evaluated according to the distribution characteristics of the data, using the "Student's t-test" or the "Wilcoxon signed rank test." Comparisons of categorical variables were made with the Chi-square or Fischer's exact test according to case distributions.

## Results

**Figure 2:**
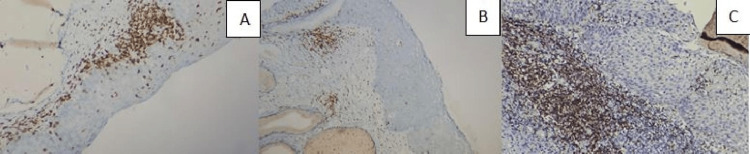
(A) CD5 in the stroma of the CIN II case, IHC, 100×, (B) CD5 in epithelium (tumoral) and stroma of the CIN III case, IHC, 200×, and (C) highly increased CD5 in the stroma of the CIN III case, IHC, 100×.

The mean age of the HSIL group was statistically significantly lower (p=0.000). There was no difference between groups in excision type and microscopic lesion size. Endocervical (EC) gland involvement was absent in the LSIL group (p=0.000). There was a statistically significant increase in the HSIL group for Ki-67 (Figure 2A), which was examined together with hematoxylin-eosin staining in the pathology archive. Baseline data on patients and a review of previous pathology data are presented in Table 1.

**Table 1:** Basic features of clinical data. LSIL: low-grade squamous intraepithelial lesion, HSIL: high-grade squamous intraepithelial lesion, CIN: cervical intraepithelial neoplasia, LEEP: loop electrosurgical excision procedure, EC gland: endocervical gland. *p ˂0.05: statistically significant.

Features		LSIL (n=30) mean ± Std	HSIL (n=30) mean ± Std	P-value
Age		52.00±9.04	40.27±8.72	0.000*
Microscopic CIN size, mm^2^		4.63±2.48	4.20±2.00	>0.05
Type of excision	LEEP	20/30 (66.6%)	16/30 (53.3%)	>0.05
	Conization	10/30 (33.3%)	14/30 (46.6%)	
EC gland involvement	Positive	0/30 (0.0%)	13/30 (43.3%)	0.000*
	Negative	30/30 (100.0%)	17/30 (56.6%)	
Ki-67		14.13±1.90	76.33±13.76	0.000*

**Figure 3:**
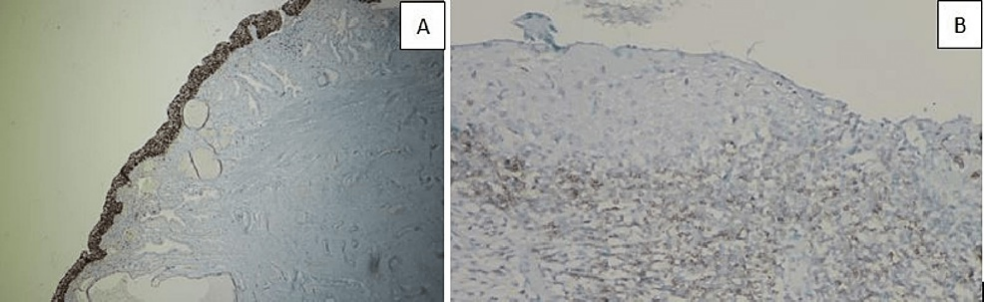
(A) Full-thickness staining for Ki-67 in the epithelium (tumoral) in case of CIN III, IHC, 100×; (B) increased PD-1 in the stroma of the CIN III case, IHC, 200×. CIN: cervical intraepithelial neoplasia, IHC: immunohistochemistry.

**Figure 4:**
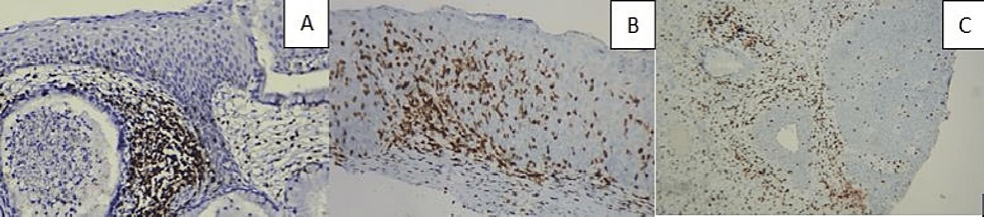
(A) Increased CD3 in the stroma of the CIN II case, IHC, 100×; (B) highly increased CD3 in the epithelium (tumoral) of the CIN III case, IHC, 200×, (C) increased CD3 in the stroma in the area of CIN III case and in the endocervical gland involvement, IHC, 200×. CIN: cervical intraepithelial neoplasia, IHC: immunohistochemistry.

**Figure 5:**
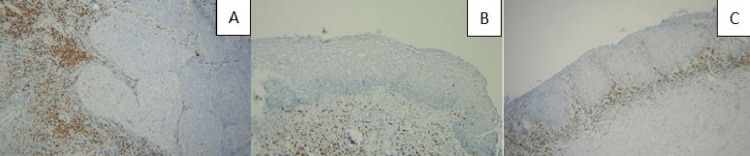
(A) Increased CD4 in the stroma, IHC, 200×; (B) CD4 in CIN I case, IHC, 100×; (C) increased CD4 in the stroma of the CIN III case, IHC, 200×. CIN: cervical intraepithelial neoplasia, IHC: immunohistochemistry.

**Figure 6:**
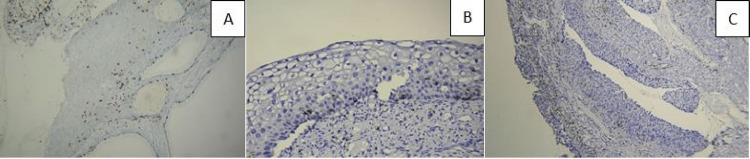
(A) CD8 in the area of CIN III and in the endocervical gland involvement, IHC, 200×; (B) CD8 in CIN I case, IHC, 200×; (C) CD8 in the areas of CIN III case and in the endocervical gland involvement, IHC, 200×. CIN: cervical intraepithelial neoplasia, IHC: immunohistochemistry.

Tumoral (epidermal) stainings had a statistically significantly higher staining rate than the determined target percentages. In the tumoral lesion area, CD3 (Figure 3), CD4 (Figure 4), CD5 (Figure 5), CD8 (Figure 6), and PD-1 (Figure 2B) were highly stained in 20/30 (66.6%), 16/30 (53.3%), 15/30 (50.0%), 24/30 (80.0%), and 13/30 (43.3%) of the cases for the HSIL group, while 7/30 (23.3%), 4/30 (13.3%), 5/30 (16.6%), 9/30 (30.0%), and 5/30 (16.6%) were in the LSIL group, respectively. In the examination of immunostaining in the stromal area, there was a statistically significant high staining in a large number of cases in the HSIL group only for PD-1 (19/30 [63.3%] vs. 6/30 [20.0%]). Intratumoral and stromal immunostaining in LSIL and HSIL are shown in Table 2.

**Table 2:** Intratumoral and stromal immunostaining in LSIL and HSIL. LSIL: low-grade squamous intraepithelial lesion; HSIL: high-grade squamous intraepithelial lesion; *p˂0.05; statistically significant.

CD3 intratumoral	Low	23/30, 76.6%	10/30, 33.3%	0.001*
	High	7/30, 23.3%	20/30, 66.6%	
CD4 intratumoral	Low	26/30, 86.6%	14/30, 46.6%	0.001*
	High	4/30, 13.3%	16/30, 53.3%	
CD5 intratumoral	Low	25/30, 83.3%	15/30, 50.0%	0.006*
	High	5/30, 16.6%	15/30, 50.0%	
CD8 intratumoral	Low	21/30, 70.0%	6/30, 20.0%	˂0.001*
	High	9/30, 30.0%	24/30, 80.0%	
PD-1 intratumoral	Low	25/30, 83.3%	17/30, 56.6%	0.024*
	High	5/30, 16.6%	13/30, 43.3%	
CD3 stromal	Low	17/30, 56.6%	10/30, 33.3%	0.069
	High	13/30, 43.3%	20/30, 66.6%	
CD4 stromal	Low	18/30, 60.0%	15/30, 50.0%	0.436
	High	12/30, 40.0%	15/30, 50.0%	
CD5 stromal	Low	18/30, 60.0%	12/30, 40.0%	0.121
	High	12/30, 40.0%	18/30, 60.0%	
CD8 stromal	Low	15/30, 50.0%	11/30, 36.6%	0.297
	High	15/30, 50.0%	19/30, 63.3%	
PD-1 stromal	Low	24/30, 80.0%	11/30, 36.6%	0.001*
	High	6/30, 20.0%	19/30, 63.3%	

While the percentages of all staining were statistically significantly higher in the HSIL group, there was no statistical difference only for stromal CD5 and CD8. Percentages of immunostaining according to CIN groups (LSIL and HSIL) are presented in Table 3.

**Table 3:** Percentages of immunostaining according to LSIL and HSIL group. LSIL: low-grade squamous intraepithelial lesion HSIL: high-grade squamous intraepithelial lesion; *p˂0.05; statistically significant.

	LSIL n=30		HSIL n=30		P-value
	Mean±Std	Median	Mean±Std	Median	
CD3 intratumoral	13.2±5.1	12.5	24.4±13.5	22.0	˂0.001*
CD4 intratumoral	5.37±3.47	5.0	10.57±7.75	10.0	0.002*
CD5 intratumoral	5.70±2.81	5.0	8.50±4.59	8.5	0.006*
CD8 intratumoral	7.67±3.64	6.0	13.87±7.14	15.0	˂0.001*
PD-1 intratumoral	0.27±0.69	0.0	1.83±4.07	0.0	0.046*
CD3 stromal	37.43±18.38	30.0	55.80±29.57	50.0	0.006*
CD4 stromal	17.30±13.18	10.0	29.23±22.75	17.5	0.017*
CD5 stromal	19.67±16.85	13.5	25.40±17.77	20.0	0.204
CD8 stromal	20.13±11.15	17.5	26.57±15.13	20.0	0.066
PD-1 stromal	1.03±2.35	0.0	3.57±4.39	2.0	0.008*

Intratumoral and stromal CD3, CD4, CD5, CD8 ratios in PD-1 high and low cases are presented in Table 4. Only for CD3 intratumoral staining was found to be low with 28/42 (66.6%) in the PD-1 low group and statistically high with 13/18 (72.2%) in the PD-1 high group (p=0.006). There was no statistically significant difference in other stainings according to PD-1 low and high staining.

**Table 4:** Intratumoral and stromal CD3, CD4, CD5, CD8 ratios in PD-1 high and low cases. *p ˂0.05; statistically significant.

		Low		High		P-value
CD3 intratumoral	PD-1 Low	28/42	66.6%	14/42	33.3%	0.006*
	PD-1 High	5/18	27.7%	13/18	72.2%	
CD4 intratumoral	PD-1 Low	30/42	71.4%	12/42	28.5%	0.232
	PD-1 High	10/18	55.5%	8/18	44.4%	
CD5 intratumoral	PD-1 Low	29/42	69.0%	13/42	30.9%	0.550
	PD-1 High	11/18	61.1%	7/18	38.8%	
CD8 intratumoral	PD-1 Low	22/42	52.3%	20/42	47.6%	0.079
	PD-1 High	5/18	27.7%	13/18	72.2%	
CD3 stromal	PD-1 Low	18/35	51.4%	17/35	48.5%	0.236
	PD-1 High	9/25	36.0%	16/25	64.0%	
CD4 stromal	PD-1 Low	21/35	60.0%	14/35	40.0%	0.357
	PD-1 High	12/25	48.0%	13/25	52.0%	
CD5 stromal	PD-1 Low	21/35	60.0%	14/35	40.0%	0.067
	PD-1 High	9/25	36.0%	16/25	64.0%	
CD8 stromal	PD-1 Low	17/35	48.5%	18/35	51.4%	0.333
	PD-1 High	9/25	36.0%	16/25	64.0%	

## Discussion

Cervical cancer is one of the most preventable and successfully curable types of cancer if detected early and managed effectively. Cervical intraepithelial lesions can regress or progress to cancer. Therefore, people with this lesion should be identified and kept under control. Immunity plays a role in the persistence or regression of the HPV virus [13]. Both innate and acquired immunity are required for clearing HPV.

Despite the absence of cytological abnormalities, HPV-positive women are referred to colposcopy as part of the HPV-based screening program [3]. Patients not reported as normal in the Pap test-based cytological screening program may be referred for colposcopy. Patients with visible cervical pathologies during routine gynecological examinations are also candidates for colposcopy. Colposcopy-guided biopsy remains the current gold standard method in the diagnosis of cervical lesions in patients with the abnormal colposcopic findings we have mentioned above [6]. Although cryotherapy or laser ablation is used in cervical lesions, a biopsy with conization or LEEP should be performed to determine the degree of the lesion. The excision method, which was unrelated to the persistence of HPV in our previous study, was also unrelated to the degree of the cervical lesion in our current study. Determining the degree of the lesion is important in patient follow-up. In addition, excisional procedures can provide information about the surgical margin and the need for further processing [3]. The persistence of HPV increases susceptibility to cancer [14]. Age was younger in the HSIL group considered predisposing for cancer in this current study and in the persistent group in our previous study [3]. The Ki-67 immunostaining showing the proliferative fraction in aggressive tumors such as breast, lung, and lymphoma assists the pathologist in distinguishing HSIL from their differential diagnosis [15]. In our study, all stainings were significantly correlated with Ki-67. Compared to other stainings in the current study, similar to our previous vulvar intraepithelial neoplasia study, Ki-67 had a higher percentage of staining in the HSIL group [16]. It is also helpful in the differential diagnosis as well as in determining the degree of the lesion in cauterized areas.

The asset of TILs in the intratumoral and stromal components of a cervical intraepithelial lesion is a significant prognostic factor. In CIN cases, the epithelium acts as an antigen and may induce an immune response. T-cells that infiltrate tumors are thought to mediate tumor-specific immune reagents [17]. T cells, especially CD4+ T cells, are the most important cells in viral protection. CD4+ T cells can activate B cells to produce an antibody that can destroy a pathogen and help CD8+ T cells mature. CD8+ cytotoxic T cells activated by cytokines and lymphokines released from CD4+ T-helper lymphocytes kill pathogen-infected or malignantly transformed host cells. PD-1 is expressed in activated T cells (including tumor-infiltrating T cells), regulatory T cells, B cells, NK cells, activated monocytes, and dendritic cells. Stimulation of PD-1 suppresses T-cell responses [18]. CD8+ cytotoxic T cells produce cytokines such as interferon-gamma (IFN-ɣ), tumor necrotizing factor (TNF)−α, and interleukin (IL)-2. Interferon-gamma production, which indicates the activation of the immune system, is increased in CIN lesions. Expression of CD4+ and CD8+ T cells and NK cells plays a role in this increase [13]. CD5 is a type of receptor molecule that signals cell growth in T cells. It also blocks T-cell interactions. CD5 is expressed in lymphocyte precursors, mature T and B cells, and is associated with the T cell receptor/CD3 complex [19]. In this current study, when we were examined according to whether PD-1 stained or not, those with CD3 intratumoral staining were significantly different. Tumor-associated antigen-presenting cells can use the PD1/PD-L pathway to control antitumor T cell responses [18]. In a lung cancer study related to PD-1 and CD5, it was stated to have an antitumor effect. This study also mentioned that CD5 and PD-1 could be used as immunotherapies in cancer treatment [19]. However, in our study, on the contrary, we showed that these two markers were high in the peri-neoplastic region in the HSIL group. Interestingly, they were less expressed in the epithelium than in the stroma.

The reduction of T-cell responses caused by the interaction between PD-1 and PD-L1 is a crucial factor in the activation and growth of autoreactive T cells. In the same study, it was stated that the expression of PD-1 in the tumoral region increased and decreased tumoral expansion with its inhibition [9]. In a study emphasizing the need for further studies to demonstrate the success of immunotherapy in cervical cancer, it was stated that PD-L1 and CD8 levels were found to be dramatically higher in these cases [20]. In our study, CD8 and PD-1 were higher in the pre-neoplastic group, which is consistent with the literature. Due to its role in PD-1/PD-L1 tumor growth, it has been stated that it can be used in the treatment of cervical cancer through DNA mismatch repair with its inhibition [9]. One of the most recent studies describing the importance of identifying biomarkers for immune checkpoint inhibitors in gynecological cancers emphasized that patients can tolerate nivolumab and pembrolizumab (PD-1 receptor blockers) with very successful outcomes [7].

Limitation

The data of the patients were insufficiently recorded. Due to the retrospective nature of the study, abnormal colposcopic findings were not recorded in all patients and could not be analyzed. In addition, unfortunately, we could not reach information such as smoking, gravida, or parity, which we think may affect the cervical intraepithelial lesion.

## Conclusions

Cervical cancer is still a very important women's health problem, especially in countries where effective screening programs cannot be implemented. Assuming that HPV acts on the immune system, showing these steps one by one will be important for solutions that can bring treatment as well as diagnosis. CD3, CD4, CD5, CD8, and PD-1 positive cells are present in the epithelium of high-grade lesions at a higher rate than in low-grade lesions. CD3, CD4, and CD8 also show a positive correlation with the Ki-67 proliferation index. In the development of immunotherapy, it is necessary to fully understand the tumoral microenvironment and all the cell pathways that occur in this area. We believe that CD3, CD4, CD5, and CD8 may contribute to PD-1-mediated tumor control. Anti-PD-1 therapy can be used, especially in cervical precancerous and cancerous PD-1-positive cases. However, larger studies are needed to evaluate the importance of this relationship in terms of prognosis.
